# The Proportion of Women Who Have a Breast 4 Years after Breast Cancer Surgery: A Population-Based Cohort Study

**DOI:** 10.1371/journal.pone.0153704

**Published:** 2016-05-05

**Authors:** Joanna C. Mennie, Pari-Naz Mohanna, Joseph M O’Donoghue, Richard Rainsbury, David A. Cromwell

**Affiliations:** 1 Clinical Effectiveness Unit, Royal College of Surgeons of England, London, United Kingdom; 2 Department of Plastic and Reconstructive Surgery, St Thomas Hospital, Guy’s and St Thomas’ NHS Foundation Trust, London, United Kingdom; 3 Department of Plastic and Reconstructive Surgery, Royal Victoria Infirmary, Newcastle Upon Tyne NHS Foundation Trust, Newcastle upon Tyne, United Kingdom; 4 Department of Breast Surgery, Royal Hampshire County Hospital, Hampshire Hospitals NHS Foundation Trust, Winchester, United Kingdom; 5 Department of Health Services Research & Policy, London School of Hygiene & Tropical Medicine, London, United Kingdom; Osaka University Graduate School of Medicine, JAPAN

## Abstract

**Background:**

There are numerous pathways in breast cancer treatment, many of which enable women to retain a breast after treatment. We evaluated the proportion of women who have a breast, either through conserving surgery (BCS) or reconstruction, at 4-years after diagnosis, and how this varied by patient group.

**Methods and Findings:**

We identified women with breast cancer who underwent initial BCS or mastectomy in English National Health Service (NHS) hospitals between January 2008 and December 2009 using the Hospital Episode Statistics (HES) database. Women were assigned into one of four patient groups depending on their age at diagnosis and presence of comorbidities. The series of breast cancer procedure (BCS, mastectomy, immediate, or delayed reconstruction) undergone by each women was identified over four years, and the proportion of women with a breast calculated. Variation was examined across patient groups, and English Cancer Networks. Between 2008 and 2009, 60,959 women underwent BCS or mastectomy. The proportion with a breast at 4 years was 79.3%, and 64.0%, in women less than 70 years without, and with comorbidities. Whilst in women aged 70 and over without, and with comorbidities, proportions were 52.6%, and 38.2%, respectively. Comorbidities were associated with lower proportions of BCS, but had little effect on reconstruction rates unlike age. Networks variation of 15% or more was found within each patient group, and Cancer Networks tended to have either a high or low proportion across all four patient groups. However, while 14% of women under 70 years had undergone reconstruction, less than 2% of women aged 70 or more had this treatment option.

**Conclusion:**

The proportion of women diagnosed with breast cancer who retain a breast at 4 years is strongly associated with age, and presence of comorbidities. There was significant variation between Cancer Networks indicating that women’s experience in England was dependent on their geographical location of treatment.

## Introduction

The psychosocial impact on women with breast cancer who undergo mastectomy has been well documented. Loss of the breast has been associated with a negative impact on confidence, emotional and sexual well-being, as well as satisfaction with appearance.[1] This effect is widely recognised, and women have various potential options to retain a breast after treatment. For women with early-stage breast cancer, evidence has shown no difference in survival benefit between breast conserving therapy (BCS) with adjuvant Radiotherapy and mastectomy alone.[2] In addition the development of breast cancer screening and earlier detection of breast cancer, alongside the developments in adjuvant therapies, have contributed to a rise in women having this type of surgery.[3]

For women undergoing mastectomy, the National Institute for Health and Clinical Excellence (NICE) has recommended that reconstruction should be available to all women since 2002.[4] However, despite this guidance, gross inequalities in access to oncoplastic reconstruction services were highlighted by the National Mastectomy and Breast Reconstruction Audit in 2008.[5,6] NICE subsequently published revised guidelines that reiterated the importance of women being offered reconstruction, irrespective of whether the service is available locally.[7]

Whether a women’s specific treatment pathway results in conserving or reconstructing the breast is dependent upon several factors: disease stage, tumour size, comorbidities, choice, and not only service quality but service availability. The multidisciplinary approach to breast cancer in the UK recognises that all such variables have to be considered, and that the selected pathway is arrived at after an informed patient choice.

However, not all women achieve their initial treatment outcomes. For example, women having BCS as their primary treatment may proceed to mastectomy if resection margins are incomplete, and in women with mastectomy and reconstruction, the reconstruction may fail. Previous studies examining breast cancer care pathways have been limited by short follow-up that would have missed these pathway changes, or limited by reporting on only one type of surgical outcome. [3,6,8]

With the multiple interconnected potential pathways, assessing the care women with breast cancer receive is complex, [9] and we sought to describe the performance of breast cancer care across England using a new approach. We determined the proportion of women who have a breast, either through conserving surgery or reconstruction, 4-years from the date of initial breast cancer surgery. We also examined whether the proportion was influenced by age or comorbidities, and whether the proportion varied across the English Cancer Networks.

## Methods

### Patient selection

This study used data extracted from the Hospital Episode Statistics (HES) database between 1 January 2000 and 31 March 2014.[10] This database contains records on all patients admitted to English NHS hospitals, and allocates patients a unique identifier that allows for longitudinal follow-up of individuals. Each record contains demographic and clinical information including diagnoses, and operative procedures. Diagnoses are coded using International Classification of Diseases, 10^th^ revision (ICD10),[11] while procedures are coded using the UK Office for Population Census and Surveys classification, 4^th^ revision (OPCS4).[12]

The study included women aged 16 years or over with breast cancer (ICD10: C50 and D05) who underwent initial mastectomy (OPCS4: B27) or BCS (OPCS4:B28 excluding B28.4) between 1 January 2008 and 31 December 2009 in English NHS hospitals. Women undergoing prophylactic surgery were excluded. Women with previous or subsequent contralateral BCS or mastectomy were also excluded. We then identified all mastectomy, BCS, and breast reconstruction procedures in NHS hospitals during the subsequent four years, as previous work had shown most delayed reconstruction procedures were performed within this timeframe. We included all types of reconstruction procedures: implants, expanders, pedicled flaps and free flaps (codes available on request). Patient age was defined as age at initial breast cancer surgery. The presence of comorbidities was based on a woman’s RCS Charlson comorbidity score,[13] with the exception of a diagnosis of breast cancer (which was removed from the list of conditions counted in the Charlson score) as all patients had this diagnosis code. Finally, each woman was assigned to one of the 28 English Cancer Networks that existed on 31 March 2012 based on the hospital provider code at initial surgery.

### Outcome definition

The proportion of women with a breast at 4 years was defined as the primary outcome measure and was determined by the pattern of surgical procedures that they underwent. The proportions of women with a breast at baseline and at intermediate times were regarded as intermediate outcomes. At baseline, women were assigned into one of three treatment categories based on their initial cancer surgery: BCS, mastectomy alone, or mastectomy with immediate reconstruction. Two further treatment categories were created for women who went on to have a delayed reconstruction or who lost their reconstruction. Women were allocated to the appropriate treatment category at 1, 2, 3, and 4 years after initial surgery based on their initial category and type of breast procedure they had undergone during the subsequent years (if any). The proportion of women with a breast at each time point included only those women with BCS or an intact reconstruction after mastectomy. Women who had undergone mastectomy alone or who had suffered a loss of reconstruction were labelled as not having retained a breast.

### Analysis

The proportions of women in the five treatment categories were calculated at baseline and the annual follow-up time points for the overall cohort and for four patient groups whose definition reflected factors known to influence treatment pathways. The four groups were: women aged less than 70 years without comorbidities (Group 1) or with comorbidities (Group 2), and women aged 70 years or older without comorbidities (Group 3) or with comorbidities (Group 4).

We then examined differences in the primary outcome (the proportion of women with a breast at 4 years) among regional Cancer Networks within each patient group. To describe the degree of systematic variation in the distribution of Network proportions (ie, the amount of variation above that expected from random fluctuations), we calculated the additive overdispersion statistic using the method of moments approach.[14] The Pearson correlation coefficient was derived to examine the variation among the Cancer Network variation across patient groups. Finally, we examined the contribution of BCS and reconstruction procedures to the proportion of women with a breast at 4 years in the individual Cancer Networks among women aged less than 70 years without comorbidities (Group 1). The statistical significance of differences in the use of BCS and reconstruction was assessed with the chi-squared test. All statistical tests were two-sided with p-values less than 0.05 indicating a significant result. The analyses were performed using STATA version 13.1.

We identified patients who died in hospital during the 4 year follow-up and investigated how removing these patients from our cohort after their death changed the results. Their removal produced only minimal differences and consequently, for simplicity, these patients were not excluded from the results presented.

### Ethics Statement

The study was exempt from UK NREC approval because it involved the analysis of an existing dataset of anonymous data for service evaluation. Approvals for the use of HES data were obtained as part of the standard Hospitals Episode Statistics approval process.

## Results

Between January 2008 and December 2009, a total of 60,959 women with breast cancer underwent primary BCS or mastectomy procedures in English NHS trusts. Women were mostly less than 70 years, of white ethnicity, with no comorbidities, and had a diagnosis of invasive disease **(S1 Appendix).** The majority of women (n = 39,193; 64.3%) underwent initial BCS (**Table 1**). Out of the 21,766 women having a mastectomy, 3084 (14.2%) received an immediate reconstruction.

**Table 1:** Number and proportion of women with BCS, Mastectomy, Mastectomy with reconstruction and loss of reconstruction at initial surgery and yearly for 4 years.

Type of surgery	Number of women at initial surgery	Initial surgery (%)	At 1 year (%)	At 2 years (%)	At 3 years (%)	At 4 years (%)
BCS	39,193	64.3	58.4	58.2	57.9	57.6
Mastectomy alone	18,682	30.6	34.4	32.7	31.6	31.4
Mastectomy with Reconstruction	3,084	5.1	6.8	8.5	9.8	10.3
Loss of Reconstruction	n/a	n/a	0.4	0.6	0.7	0.7

After 4 years, the number of women still categorised as BCS had dropped from 64.3% to 57.6%. The drop occurred predominantly in the first year of follow-up, with 3,587 women proceeding to mastectomy (with or without reconstruction). Of these women, 82.7% underwent a mastectomy within 90 days. The proportion of women in the “mastectomy alone” category changed in each year, reflecting both the number of mastectomies after BCS and women having delayed reconstruction. At 4 years, a total of 6284 women had undergone reconstruction (immediate or delayed) after mastectomy, which represents 24.6% of all women who had mastectomy, and 10.3% of the total cohort. We identified 439 women whose reconstruction after mastectomy had failed and who had not undergone further reconstruction at 4 years follow-up. Overall, the proportion of women with a breast 4 years after initial breast cancer surgery was 67.9%.

**Fig 1:**
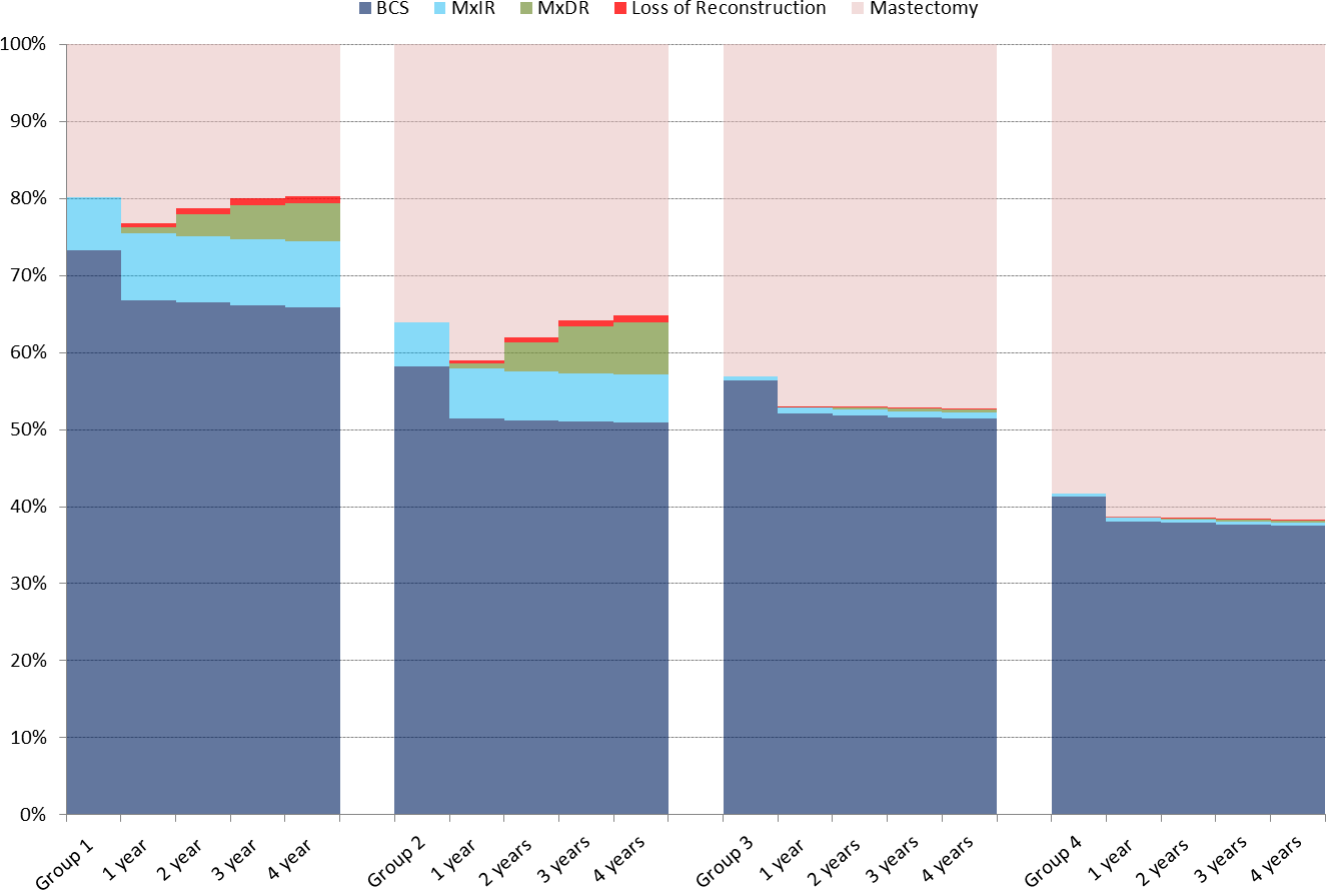
Proportion of women with BCS, MxIR, MxDR, Loss of Reconstruction, and Mastectomy at 1 year intervals after initial surgery in each patient group. MxIR: Mastectomy with immediate reconstruction. MxDR: Mastectomy with delayed reconstruction.

There were distinct differences between the four patient groups in the proportion of women with a breast at 4 years (**Fig 1**). In women aged less than 70 years without comorbidities (Group 1), 79.3% of women retained a breast at 4 years. In women of the same age but with comorbidities (Group 2), 64% of women had a breast 4 years. Among women aged 70 years and over, the proportion of women with a breast at 4 years was 52.6% and 38.2%, respectively, when comorbidities were not present / present.

This difference between the groups was predominantly related to a difference in BCS proportions rather than to the use of reconstruction. For example, in Group 1, 65.9% of women maintained a breast through BCS, whereas for women in Group 2, this proportion was only 50.9%. Few women aged 70 years and over underwent reconstruction.

Whilst the overall proportion of women with a reconstructed breast in Groups 1 and 2 did not differ, there were differences in the ratio of immediate to delayed reconstructions. Of the women with a reconstructed breast in Group 1, 63.8% had immediate reconstruction, and 36.2% delayed reconstruction. In Group 2, the proportions were 47.9% (immediate) and 52.1% (delayed).

**Fig 2:**
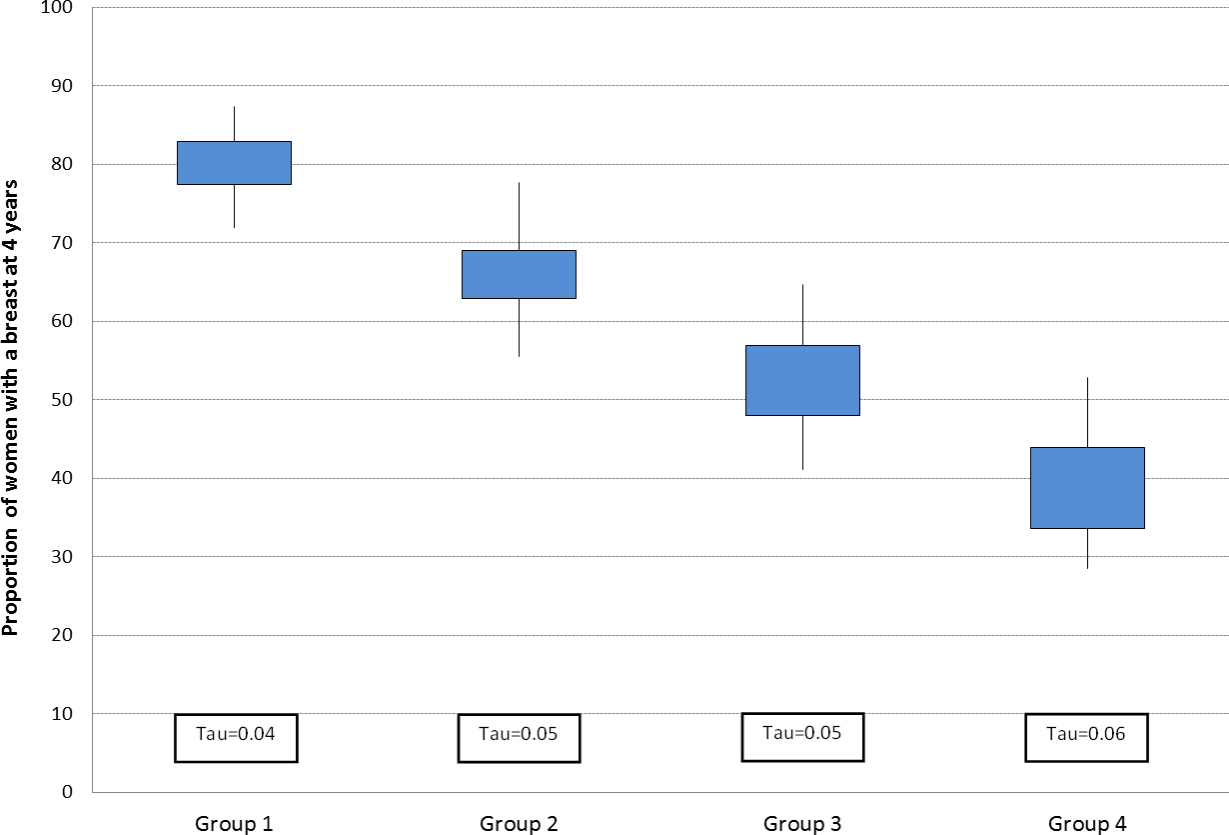
Distribution across Cancer Networks in the proportion of women with a breast 4 years after initial cancer surgery for each patient group. The bottom and top of the box show the 25^th^ and 75^th^ percentiles, and the bottom and top lines show the minimum and maximum values, respectively. The degree of systematic variation within each group is described using an additive overdispersion statistic, tau, the square root of the between-Network variance.

There was substantial variation across Cancer Networks in the proportion of women with a breast at 4 years within each patient group (**Fig 2**). There was at least at 15% absolute difference between the highest and lowest Network values within each group, with the least variation being observed in the younger, most fit women (Group 1). The greatest Network-level variation was found among women aged 70 years or older with comorbidities (Group 4). In this group of women, the proportion of women with a breast ranged from 28.6% to 52.9%.

**Fig 3:**
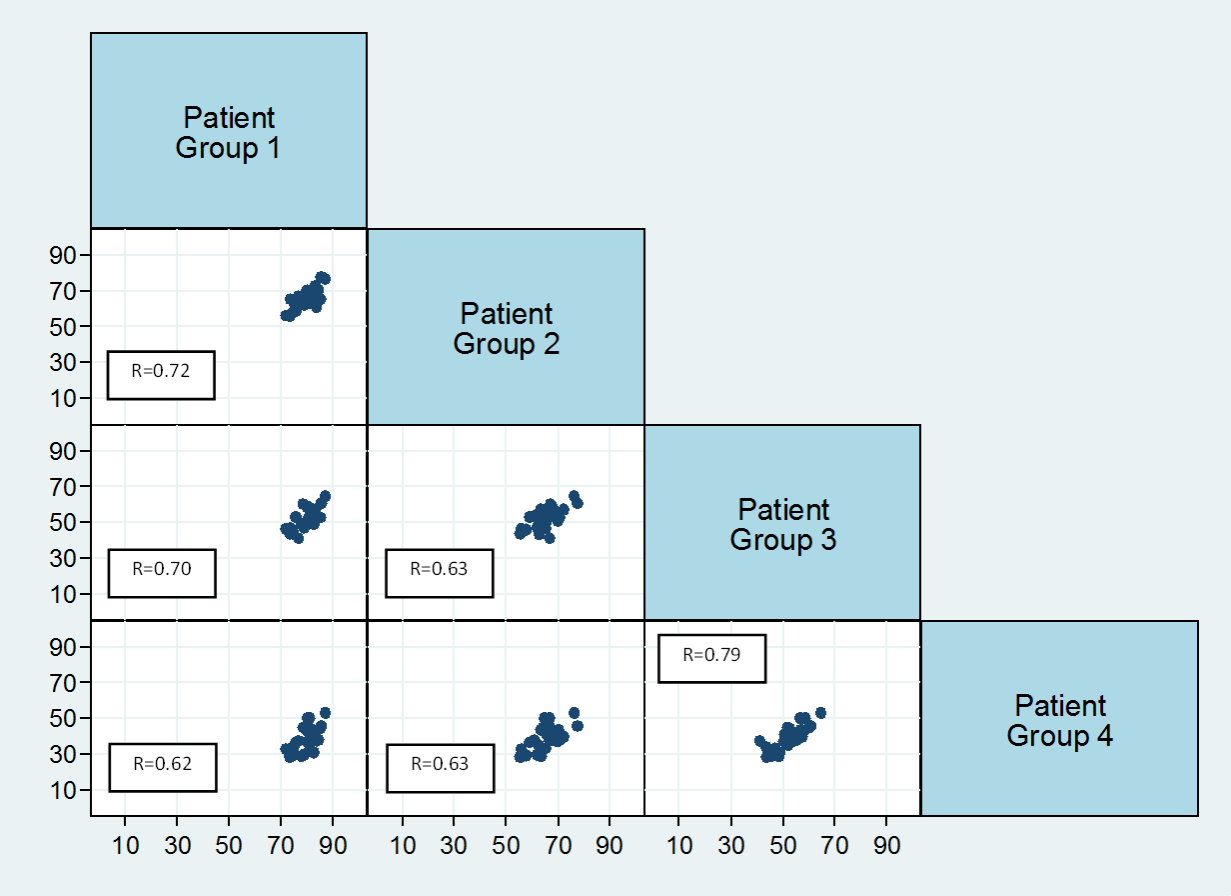
Correlation matrix showing correlation between the proportions of women with a breast in each patient group across the Cancer Networks. The x-axis represents the group at the top of the plot, and the y-axis the group on the right. The linear association between patient groups is described by the correlation coefficients.

The scatter plots in **Fig 3**reveal that Networks with a high proportion of women with a breast after 4-years in one group tended to have high values across the other patient groups. There were some exceptions **(S2 Appendix)**. One Network had a relatively low proportion of women with a breast for Group 1, but a relatively high proportion of women with a breast across Groups 2–4. Another Cancer Network had one of the highest proportions of women with a breast in Group 1, but as comorbidities and age increased, their values dropped to become one of the lowest amongst all Cancer Networks.

**Fig 4:**
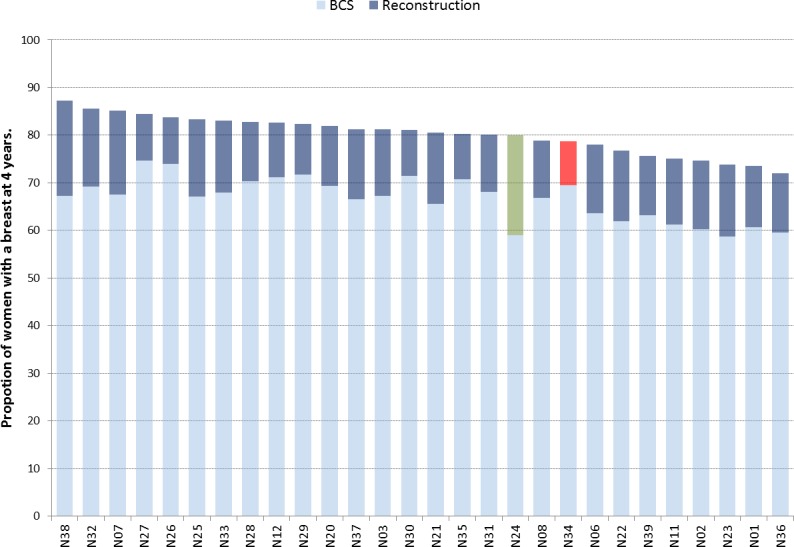
Variation across Cancer Networks of the proportion of women with a breast 4 years after initial cancer surgery through BCS and Reconstruction in patient group 1.

We examined the ratio of BCS and reconstruction procedures among patients in which it was expected that there would be the most uniform pattern of care: women aged less than 70 years without comorbidities (Group 1). There were significant differences in the ratio of BCS to reconstruction across the Networks (chi-squared test = 207.7, p<0.001), and there did not seem to be a typical ratio of BCS to reconstruction (**Fig 4)**. For example, Cancer Networks N24 and N34 had a similar proportion of women with a breast at 4-years but Network N34 achieved this with a relatively high proportion of BCS (and the lowest reconstruction rate at 9.2%) while Network N24 had the highest proportion of reconstructions at 21% and a relatively low proportion of women with BCS. **(S3 Appendix)**

## Discussion

Many studies use the rate of breast conserving surgery among women with breast cancer or the rate of reconstruction after mastectomy as measures of performance for breast cancer units. However, these measures fail to capture the complexity of the treatment pathway and so lead to only a partial understanding of practice. In this study, we have adopted the proportion of women with a breast at 4 years after their initial surgery as an alternative, more comprehensive measure. The measure includes breast conserving, mastectomy, and reconstruction procedures and thus provides a more reliable picture of clinical practice. It is appreciated that due to intricacies such as touch and feel that BCS is not equivalent to reconstruction. However both BCS and Reconstruction procedures result in a better quality of life and patient reported breast satisfaction comparative to mastectomy alone.[15,16]

Overall, we found that 67.9% of 60,959 women undergoing initial breast cancer surgery in English NHS hospitals had a breast after 4 years. But grouping women according to their age and the presence of comorbid conditions revealed important differences between patients. The proportion of women with a breast after 4 years was highest (79.3%) among women aged less than 70 years without comorbidities. For comorbid women in the same age band, the proportion had dropped to 64.0%. Among women aged 70 years or older, the proportion was 52.6%, and 38.2%, respectively for women with and without comorbidities. The proportions varied substantially among the 28 Cancer Networks within each of the patient groups, with this variation being most significant for comorbid women aged 70 years or older. In addition, we found that Cancer Networks tended to have a high / low proportion consistently across all four patient groups. Although these Networks no longer exist, they still represent an informative level of aggregation as the provision of breast conserving, mastectomy, and reconstruction procedures are interconnected within English regions.

### Strengths and limitations

The study has several strengths. First, it used a comprehensive national database that included all women undergoing initial breast cancer surgery over a 2-year period in English NHS hospitals, which reduced the risk of selection bias and yielded a large study cohort. Second, the follow-up period for each patient was 4 years, which exceeded the mean time to delayed reconstruction reported in literature, and allowed pathway changes to be identified. [17,18]

The study has a number of limitations. The coding of diagnoses and procedures in an administrative hospital database may suffer from errors or omissions. Studies examining the coding of breast cancer surgery within HES, however, concluded that the coding of these procedures is accurate, finding 90–93% agreement with data provided by surgeons in England.[19]Moreover, there are specific codes for breast conserving surgery and mastectomy, which should reduce the risk of mis-classifying operations. We used a wide range of procedure codes to identify reconstruction and loss of reconstruction in order to minimise the effect of different coding patterns within hospitals. Consequently, bias due to errors in procedure coding is likely to be small.

Another limitation of HES is that it does not contain information on tumour size, disease stage, or patient preference. The decision for BCS versus mastectomy, and the proportion of women with a conserved breast, will be influenced by these variables. The National Cancer Intelligence Network has reported only modest variation in breast cancer incidence between regions.[20] Further, two separate studies have shown non-significant variation in breast cancer stage at presentation across Cancer Network regions.[21,22] Adjusting for tumour size or disease stage would therefore be unlikely to significantly reduce the between-Network variation that was observed.

HES also does not contain information on whether the cancer was screen-detected or symptomatic. During the study time period (January 2008 to December 2009), routine breast cancer screening was offered to women aged 50–70 years with an uptake of 77%.[23] A review of practice in the UK found significantly higher BCS rates in women with screen-detected cancers (73%) versus symptomatic cancers (47%), which may explain the influence of age on BCS proportions.[24] Nonetheless, differences in the presentation route between women aged less than and greater than 70 years would not account for the significant variation observed in the use of reconstruction.

### Clinical implications

The findings of this study highlight two issues that influence the proportion of women having surgery for breast cancer who retain a breast at 4-years. The first is whether the initial surgery undergone was BCS or mastectomy, despite the option of immediate or delayed reconstruction. The second is the influence of increasing age and the presence of comorbidities on the type of surgery undergone.

Increasing age and the presence of comorbidities might be expected to lower the ratio of BCS to mastectomy for various reasons.[9] For instance, whilst survival following BCS with radiotherapy has been shown comparable to mastectomy, a woman may require further surgery after BCS due to compromised margins.[2] In comorbid women, the higher proportion of mastectomies may reflect a perceived benefit of having one operation and an attempt to avoid further anaesthetic risk or radiotherapy. However Eaker et al found large differences across the whole pathway of care in older women, and concluded that it would be difficult to explain by comorbidities alone.[8] It should not be assumed that the overall proportion of women with a breast at 4 years within Groups 2–4 cannot be increased. It has been shown that women from deprived areas, who are more likely to have comorbidities, are less likely to attend for screening.[25] Current differences in the presentation route amongst comorbid women may therefore impact on a woman’s suitability for BCS, and these could be amenable to targeted improvement initiatives.[8,9]

Another area for further exploration is the influence of comorbidity on the ratio of immediate to delayed reconstruction. Oncoplastic breast reconstruction guidelines recommend reconstruction should be offered to all women expected to have a mastectomy, except where comorbidities preclude it.[26] The results of this study are broadly consistent with this recommendation among women aged less than 70 years, with 63.8% of reconstructions being immediate in women without comorbidities compared with 47.9% in women with comorbidities. However, there may be further potential to increase the proportion of women retaining a breast with greater use of delayed reconstruction.

Our study again highlights low rates of reconstruction among women aged over 70 years, even without precluding comorbidities, something that was reported in a review of UK practice in 2007.[24] One explanation for this finding is that advanced tumours that require adjuvant therapy are more common among older women and, as such, a lower percentage of these women will be suitable for immediate reconstruction;[27] oncoplastic guidelines recognise the decision for immediate reconstruction should be made in consideration for potential adjuvant therapy.[26] However, the 4-year follow-up of our study allows sufficient time for women potentially requiring adjuvant therapy to undergo delayed reconstruction and so it seems unlikely that this explanation accounts for these findings. More plausible explanations for the low rate of reconstruction in older women are patient preferences and/or restrictions on access.[28]

That there is potential to increase the proportion of women retaining a breast is best demonstrated by the variation we observed across Cancer Networks, and in particular that Networks seemed to perform consistently so across all four patient groups. This suggests that the structure and process by which care is delivered in these Networks play an important role in determining the specific pathway followed by women, and both require investigation.[29] Moreover, while the variation across Cancer Networks was greatly influenced by the proportion of women receiving BCS, there was not a uniform picture across the networks in terms of the use of BCS and reconstruction. Among Networks with a high proportion of women having BCS, there were Networks with both high and low proportions of women having mastectomy with reconstruction. This reiterates the importance of reporting BCS, mastectomy, and reconstruction together when investigating the provision of breast cancer services.

## Conclusion

In this study, we used the proportion of women with a breast at 4 years after their initial surgery as comprehensive measure of breast cancer surgery to describe the complex outcomes of breast cancer surgery in a simple way and which provides useful information for patients and health services. Overall, we found that two-thirds of women undergoing breast cancer surgery in English NHS hospitals retain a breast after 4 years. However, this proportion was strongly influenced by age and comorbidities, with the proportions in our four patient groups being 79.3%, 64.0%, 52.6%, and 38.2%. In addition, we found significant variation across Cancer Networks in all four patient groups. This is of concern as it indicates that women’s experience and standard of care is dependent on their geographical location of treatment. These results should encourage breast cancer services to review their performance with the aims of both reducing the regional variation and increasing access to BCS and post-mastectomy reconstruction.

## Supporting Information

S1 Appendix Cohort’s demographics. (DOCX)

S2 Appendix Variation across Cancer Networks in each patient group. Variation across Cancer Networks of the proportion of women with a breast 4 years after initial cancer surgery in each patient group. Networks are ordered based on proportion of women with a breast in Group 1 from low to high. Red equates to low volumes, and blue to high volumes.
(DOCX)

S3 Appendix Cancer Network Index. (DOCX)

## Abbreviations

BCS: Breast conserving surgery
HES: Hospital Episode Statistics
ICD10: International Classification of Diseases, 10^th^ revision
NICE: National institute for clinical excellence
OPCS4: Office for Population Census and Surveys classification, 4^th^ revision
