# Supplementary material for: The Proportion of Women Who Have a Breast 4 Years after Breast Cancer Surgery: A Population-Based Cohort Study
Source: PLoS One. 2016 May 5;11(5):e0153704. doi: 10.1371/journal.pone.0153704 (PMC4858207; doi:10.1371/journal.pone.0153704)
Supplement: S1 Appendix — (DOCX) [file pone.0153704.s001.docx]

| **Variable** | | **Number of women** | **Proportion of women** |
| --- | --- | --- | --- |
| ***Age*** | *<70 years* | 45,913 | **75.3%** |
|  | *>70 years* | 15,046 | **24.7%** |
| ***Number of comorbidities*** | *0* | 41,432 | **68.0%** |
|  | *1* | 16,078 | **26.4%** |
|  | *>1* | 3449 | **5.7%** |
| ***Disease*** | *DCIS* | 5,677 | **9.3%** |
|  | *Invasive* | 51,077 | **83.8%** |
|  | *Invasive +DCIS* | 4,205 | **6.9%** |
| ***Ethnicity*** | *White* | 54,535 | **89.5%** |
|  | *Asian* | 2,737 | **4.5%** |
|  | *Black* | 1,231 | **2.0%** |
|  | *Unknown* | 2,456 | **4.0%** |
| *IMD* | *1 (least deprived)* | 13,661 | **22.4%** |
|  | *2* | 13,872 | **22.8%** |
|  | *3* | 12,946 | **21.3%** |
|  | *4* | 11,101 | **18.2%** |
|  | *5 (most deprived)* | 9,144 | **15.0%** |
|  | *Unknown* | 235 | **0.4%** |

***S1 Appendix:*** *Cohort’s demographics.*
