# Supplementary material for: The Proportion of Women Who Have a Breast 4 Years after Breast Cancer Surgery: A Population-Based Cohort Study
Source: PLoS One. 2016 May 5;11(5):e0153704. doi: 10.1371/journal.pone.0153704 (PMC4858207; doi:10.1371/journal.pone.0153704)
Supplement: S2 Appendix — Variation across Cancer Networks of the proportion of women with a breast 4 years after initial cancer surgery in each patient group. Networks are ordered based on proportion of women with a breast in Group 1 from low to high. Red equates to low volumes, and blue to high volumes. (DOCX) [file pone.0153704.s002.docx]

***S2 Appendix:*** *Variation across Cancer Networks of the proportion of women with a breast 4 years after initial cancer surgery in each patient group. Networks are ordered based on proportion of women with a breast in Group 1 from low to high. Red equates to low volumes, and blue to high volumes.*

| **Network** | **Group 1**  **Breast Proportion %** | **Group 2**  **Breast Proportion %** | **Group 3**  **Breast Proportion %** | **Group 4**  **Breast Proportion %** |
| --- | --- | --- | --- | --- |
| *N36* | 72.0 | 56.1 | 46.3 | 32.7 |
| *N01* | 73.5 | 55.5 | 43.9 | 28.6 |
| *N23* | 73.7 | 65.1 | 46.9 | 33.1 |
| *N02* | 74.6 | 57.8 | 46.2 | 29.5 |
| *N11* | 75.1 | 63.1 | 43.6 | 34.1 |
| *N39* | 75.7 | 59.1 | 53.2 | 36.2 |
| *N22* | 76.8 | 66.9 | 41.2 | 37.4 |
| *N06* | 77.9 | 63.8 | 48.8 | 29.1 |
| *N34* | 78.7 | 67.3 | 60.2 | 44.9 |
| *N08* | 78.9 | 62.1 | 47.2 | 29.5 |
| *N24* | 79.9 | 70.2 | 50.6 | 36.6 |
| *N31* | 80.1 | 67.9 | 58.4 | 42.9 |
| *N35* | 80.2 | 66.9 | 58.8 | 50.0 |
| *N21* | 80.6 | 64.4 | 51.9 | 44.5 |
| *N30* | 81.1 | 65.0 | 56.7 | 50.2 |
| *N37* | 81.3 | 62.9 | 52.2 | 34.8 |
| *N03* | 81.3 | 65.9 | 50.7 | 40.8 |
| *N20* | 82.0 | 70.4 | 52.0 | 43.4 |
| *N29* | 82.3 | 63.4 | 57.1 | 43.6 |
| *N12* | 82.7 | 62.9 | 48.9 | 30.9 |
| *N28* | 82.8 | 68.4 | 53.4 | 42.4 |
| *N33* | 83.0 | 69.8 | 55.5 | 38.1 |
| *N25* | 83.3 | 72.5 | 57.3 | 39.6 |
| *N26* | 83.7 | 61.0 | 53.4 | 37.6 |
| *N27* | 84.5 | 70.7 | 52.6 | 37.9 |
| *N07* | 85.1 | 65.3 | 52.6 | 44.3 |
| *N32* | 85.5 | 77.6 | 60.6 | 45.6 |
| *N38* | 87.3 | 76.4 | 64.7 | 52.9 |
