# Supplementary material for: The Proportion of Women Who Have a Breast 4 Years after Breast Cancer Surgery: A Population-Based Cohort Study
Source: PLoS One. 2016 May 5;11(5):e0153704. doi: 10.1371/journal.pone.0153704 (PMC4858207; doi:10.1371/journal.pone.0153704)
Supplement: S3 Appendix — (DOCX) [file pone.0153704.s003.docx]

| **Cancer Network** | **Network Name** |
| --- | --- |
| N01 | Lancashire and South Cumbria |
| N02 | Greater Manchester |
| N03 | Merseyside and Cheshire |
| N06 | Yorkshire |
| N07 | Humber and Yorkshire Coastal |
| N08 | North Trent |
| N11 | Pan Birmingham |
| N12 | Arden |
| N20 | Mount Vernon |
| N21 | West London |
| N22 | North London |
| N23 | North East London |
| N24 | South East London |
| N25 | South West London |
| N26 | Peninsula |
| N27 | Dorset |
| N28 | Avon, Somerset and Wiltshire |
| N29 | 3 Counties |
| N30 | Thames Valley |
| N31 | Central South Coast |
| N32 | Surrey, West Sussex and Hampshire |
| N33 | Sussex |
| N34 | Kent and Midway |
| N35 | Greater Midlands |
| N36 | North of England |
| N37 | Anglia |
| N38 | Essex |
| N39 | East Midlands |

***S3 Appendix:*** *Cancer Network Index.*
